# Reconciling differences between EUCAST ‘breakpoint-defining doses’ and UK national antimicrobial treatment guidelines

**DOI:** 10.1093/jac/dkag315

**Published:** 2026-09-08

**Authors:** Conor Jamieson, Sara E Boyd, Mandy Wootton, David M Livermore, Kieran Hand, Alasdair P Macgowan, Robin A Howe, BSAC Standing Committee on Antimicrobial Susceptibility Testing, Sara Boyd, Nicholas Brown, Phillipa Burns, Pegah Hafezi, Robin Howe, Stephen Hughes, Lim Jones, David Livermore, Alasdair Macgowan, Mairi Macleod, Christopher Teale, Mandy Wootton

**Affiliations:** Medical Directorate, NHS England (Midlands), Birmingham, UK; David Price Evans Global Health and Infectious Disease Research Group, University of Liverpool, Institute of Systems, Molecular & Integrative Biology, William Henry Duncan Building, Liverpool L7 8TX, UK; Infection Clinical Academic Group, St. George’s Hospital NHS Foundation Trust, Blackshaw Road, London SW17 0QT, UK; Specialist Antimicrobial Chemotherapy Unit, Public Health Wales, University Hospital of Wales, Cardiff, UK; Norwich Medical School, University of East Anglia, Floor 2, Bob Champion Research & Educational Building, James Watson Road, Norwich NR4 7UQ, UK; Medical Directorate, NHS England, London, UK; Antimicrobial Reference Laboratory, North Bristol NHS Trust, Westbury-on Trym, Bristol, UK; Specialist Antimicrobial Chemotherapy Unit, Public Health Wales, University Hospital of Wales, Cardiff, UK

## Abstract

The British National Formulary and the NICE provide empirical treatment guidance for the management of various infections, including recommendations on antibiotic doses. In parallel, and for over 15 years, EUCAST has published details of the doses upon which their antimicrobial susceptibility breakpoints are predicated, termed here ‘breakpoint-defining doses’. Recently, differences between these dosing recommendations have attracted attention, particularly since EUCAST introduced its ‘susceptible, increased exposure’ category of susceptibility. We discuss where and why these differences exist and how to reconcile them.

Guidance from the British National Formulary (BNF) and the NICE provides empirical treatment recommendations for the syndromic management of various clinical conditions, including infections.^1^ This guidance is typically rooted in the posology described in a medicine’s Summary of Product Characteristics (SmPC). The BNF/NICE guidelines indicate the antimicrobial dosages expected to give a high likelihood of clinical success, balanced against potential adverse effects.^2^ The recommendations rarely relate to specific pathogens, but doses are often adjusted in relation to the severity of infection or the patient’s underlying condition and organ function. In contrast, EUCAST’s approach is to define antimicrobial susceptibility testing (AST) breakpoints that integrate (i) MIC distributions from laboratories across the world, (ii) a knowledge of mechanisms of resistance and their effects on MICs, (iii) pharmacokinetic (PK)–pharmacodynamic (PD) modelling and (iv) clinical outcome and toxicology data. Used together with standardized reproducible testing, these are predictive, within statistical limits, of clinical outcomes for the majority of infections caused by a particular pathogen or group of pathogens.^3^

EUCAST’s tabulated ‘dosages used to define breakpoints’ can be thought of as ‘breakpoint-defining doses’ but should not be considered a table of routinely recommended empirical doses for clinical practice.^4^ This is made explicit in their table header indicating that ‘dosages can vary widely by indication’ and their guidance that this table ‘does not replace specific national, regional or local dosing guidance’.^4^ Nevertheless, confusion on this aspect is widespread, arising partly because, since 2019, EUCAST has defined a category of ‘susceptible, increased exposure’ and has indicated corresponding antibiotic dosages.

Historically, susceptibility was categorized as (S) susceptible, (I) intermediate or (R) resistant, with the (I) category open to multiple potential interpretations, including as a buffer zone for isolates with uncertain and borderline results.^3^ EUCAST’s 2019 redefinition narrowed the (I) category to specifically mean isolates that should prove susceptible *provided increased antibiotic exposure is achieved*, typically by administering a higher dose, prolonging the infusion time or increasing the dosing frequency. This applies, *inter alia*, for wild-type isolates of some species without any acquired resistance mechanism—for example, *Pseudomonas aeruginosa*, where typical isolates, with modal MICs, count as ‘I’ for many β-lactams, including ceftazidime, imipenem and piperacillin/tazobactam.^4^

A further issue is that EUCAST’s breakpoint-defining doses sometimes exceed the licensed dose in the SmPC or as listed in the BNF/NICE guidance (Table 1). On the other hand, the BNF/NICE- or SmPC-advocated dose ranges occasionally exceed EUCAST’s highest breakpoint-defining doses (Table 2). A final complexity is that the breakpoint-defining doses are often based on those commonly used across Europe and may differ from those recommended in the UK. For example, oral amoxicillin doses were observed to be lower in English national guidelines for four different clinical scenarios when compared with those advocated in France, Germany and Italy.^5^ Such differences predominately relate to older agents, where licensing studies and PK-PD evaluation are less robust than for newer agents. Nevertheless, it can complicate matters for newer agents when these incorporate an older agent as, e.g., in a β-lactam-β-lactamase inhibitor combination, with different licensed doses, or infusion regimens, for the β-lactam alone or in its new combination.

**Table 1.** Examples where the EUCAST breakpoint-defining doses exceed the highest SmPC-licensed dose/dose range or BNF guidance

Antibiotic class/drug class/drug	Dosages	EUCAST breakpoint-defining dose	BNF adult dosing range	SmPC licensed dose/dose range
Amikacin	Standard dosage	25–30 mg/kg q24h iv	15–22.5 mg/kg q24h iv	15–22.5 mg/kg q24h iv
Gentamicin	Standard dosage	6–7 mg/kg q24h iv	5–7 mg/kg q24h iv	3–5 mg/kg q24h iv
Tobramycin	Standard dosage	6–7 mg/kg q24h iv	1–1.67 mg/kg q8h iv	1–1.67 mg/kg q8h iv
Amoxicillin-clavulanic acid	Standard dosage	1.2 g q8h-q6h iv 625 mg q8h orally	1.2 g q8h iv 375–625 mg q8h orally	1.2 g q8h iv 375–625 mg q8h orally
	High dosage	2.2 g q8h iv 1 g q8h orally	2.2.g q8h iv 1 g q8h orally	2.2 g q8h iv 1 g q8h orally
Piperacillin/tazobactam	Standard dosage	4.5 g q6h iv by 30-min infusion^a^ Or 4.5 g q8h iv by 4-h infusion	4.5 g q8h iv by 30-min infusion	4.5 g q8h iv by 30-min infusion
	High dosage	4.5 g q6h iv by 4-h infusion	4.5 g q6h iv	4.5 g q6h iv

^a^For infections arising from complicated UTI, intra-abdominal infection and diabetic foot infections.

**Table 2.** Examples where higher doses listed by EUCAST are within the dosing range advised by NICE/BNF or as listed in the SmPC

Antibiotic class/drug class/drug	Dosages	EUCAST breakpoint-defining dose	BNF adult dosing range	SmPC licensed dose/dose range
Benzylpenicillin	High dosage	1.2 g q6h or 1.2 g q4h iv	2.4 g q6h iv	2.4–43.2 g per 24 h in 4–6 divided doses
Ampicillin	High dosage	2 g q6h iv	2 g q6h iv	2 g q6h iv
Ceftazidime	High dosage	2 g q8h or 1 g q4h iv	2 g q8h iv	2 g q8h iv
Chloramphenicol	High dosage	2 g q6h iv/orally	25 mg/kg q6h iv	25 mg/kg q6h iv

Optimizing dosage based on the breakpoint-defining dose seems most appropriate to consider when there is concern regarding the clinical progress of the patient and a pathogen has been identified from an infection, precisely because AST results are interpreted using EUCAST clinical breakpoints. Prior to identification of a pathogen, it seems reasonable to follow the BNF/NICE guidance for empirical syndromic treatment guidance which, as noted earlier, is informed by severity of infection based on clinical assessment and doses used in clinical trials. An exception is that local guidelines may recommend treatment with higher doses where particular pathogens, or outbreak strains, are likely (but not yet confirmed) and are known to have reduced susceptibility (higher MIC). For example, higher empirical doses of ceftriaxone (e.g. 4 g q24h) may be advocated in outpatient parenteral antibiotic therapy pathways for cellulitis to reliably cover *Staphylococcus aureus*. Likewise, higher empirical doses of piperacillin/tazobactam (e.g. 4.5 g q6h) arguably should be routine for nosocomial pneumonia in intensive care settings, where *P. aeruginosa* is more likely to be implicated. High doses are not, however, required for all patients. This may be most acutely recognized for the aminoglycosides, where ototoxicity and nephrotoxicity have been shown to be dose-dependent,^6,7^ and regimens that incorporate higher dosing, for example, EUCAST’s breakpoint-defining doses for amikacin (Table 1), demand a careful risk/benefit assessment. They should be used cautiously with careful, individualized, risk assessment. The complexities of decision making and local guideline development underpin the vital need for communication between the laboratory, infection specialists and clinical teams. Effective laboratory liaison integrated within antimicrobial stewardship programmes must ensure patient safety and high-quality care. This need will continue, given that EUCAST continues to update breakpoints and breakpoint-defining doses as new evidence becomes available.

In summary, the breakpoint-defining doses stated by EUCAST are for specific pathogens and advise on the antimicrobial exposure associated with a high probability of treatment success by attaining PK-PD targets for these specific pathogens and their expected MIC distribution. However, they are not intended as rigid treatment guidance as they do not take account of the severity of infection, potential adverse effects, drug interactions or other specific clinical circumstances such as immunocompromise or renal function. Infection specialists should refer to and consider EUCAST dosing tables when a specific pathogen is highly likely or has been identified and the AST results are known. If a pathogen causing infection is identified, the patient’s empirical regimen should be reviewed by the clinical team, supported by the laboratory and infection specialists. Whether or not to then change the dosage (if the same antibiotic is to be continued) should be a clinical decision guided by an assessment of the patient’s condition and organ function, infection syndrome, response to current therapy and remaining duration of the intended course. Any decision to increase the dosage should be weighed against the potential risk of adverse effects and *Clostridioides difficile* infection. Any decision not to increase the dosage, where suggested by the laboratory findings, should be weighed against the potential for sub-optimal outcome and/or selection of resistant sub-populations.

Where discrepancies exist between EUCAST’s breakpoint-defining doses and national dosing guidance (such as for NICE/BNF), we suggest agreements should be reached between relevant stakeholders, including national AST committees, microbiology laboratories and infection specialists. Whilst we focus on issues relevant to the UK, other countries may wish to consider similar assessments of EUCAST breakpoint-defining doses and their own national treatment guidelines. The aim should be to offer optimized empirical treatment advice in local guidelines informed by local or national surveillance data, likely infection aetiology and patient factors. In the UK, empirical guidelines should typically follow BNF/NICE recommendations and be in line with licensed doses in the SmPC. It is also recommended that BNF/NICE continually review their data to ensure that prescribing recommendations remain clinically appropriate and in-line with the best evidence on clinical outcomes. This is especially pertinent where (as with amoxicillin above) UK dosage guidance appears to be an outlier. It may be that further clinical trials are required to address these issues, recognizing that these are likely to need to be publicly funded, as many older drugs are off-patent. Lastly, to resolve disagreements, we would urge continuing collaboration between EUCAST, the BSAC AST Standing Committee and the Joint Formulary Committee responsible for the content of the BNF, the NICE and NHS bodies.

## Transparency declarations

S.E.B.: research support from Roche Pharma, educational support from Advanz Pharma, consultancy and/or speaker fees from Sumitovant, Shionogi, the UK Clinical Pharmacy Association, bioMérieux and Meiji Seika Pharma. A.P.M.: holds research grants/activities with Merck, Shionogi, InfectoPharm, GSK, Roche, BioVersys, iFAST, Oxford Drug Design, Bicycle Therapeutics, PACE, JPIAMR and NIHR. The other authors have nothing to declare. D.M.L.: advisory boards or *ad hoc* consultancy: AdjuTec, AstraZeneca, Centauri, GenPax, GSK, Lipovation, Meiji, Paion/Vistris, Shionogi, Sumitovant, Thermo Fisher, Uniphar and Wockhardt; paid lectures: bioMérieux, EuMedica, Pfizer, Shionogi and Zuellig Pharma; relevant shareholdings or options: GenPax, GSK, Merck & Co. and Revvity amounting to less than 10% of portfolio value. D.M.L. also has shareholdings in Arecor, Genincode, Probiotix, Rua Life Sciences, SkinBiotherapeutics and VericiDx (all with research/products pertinent to medicines or diagnostics) through Enterprise Investment Schemes, but has no authority to trade these holdings directly.
